# Characterizing Visual Fields in *RPGR* Related Retinitis Pigmentosa Using Octopus Static-Automated Perimetry

**DOI:** 10.1167/tvst.11.5.15

**Published:** 2022-05-16

**Authors:** Thomas M. W. Buckley, Amandeep Singh Josan, Laura J. Taylor, Jasleen K. Jolly, Jasmina Cehajic-Kapetanovic, Robert E. MacLaren

**Affiliations:** 1 Nuffield Laboratory of Ophthalmology, Nuffield Department of Clinical Neurosciences, University of Oxford, Oxford Biomedical Research Centre, Oxford, UK; 2 Oxford Eye Hospital, Oxford University Hospitals NHS Foundation Trust, Oxford, UK

**Keywords:** Octopus, RPGR, retinitis pigmentosa, visual field, perimetry

## Abstract

**Purpose:**

Peripheral visual fields have not been as well defined by static automated perimetry as kinetic perimetry in *RPGR*-related retinitis pigmentosa. This study explores the pattern and sensitivities of peripheral visual fields, which may provide an important end point when assessing interventional clinical trials.

**Methods:**

A retrospective observational cross-sectional study of 10 genetically confirmed *RPGR* subjects was performed. Visual fields were obtained using the Octopus 900 perimeter. Interocular symmetry and repeatability were quantified. Visual fields were subdivided into central and peripheral subfields for analysis.

**Results:**

Mean patient age was 32 years old (20 to 49 years old). Average mean sensitivity was 7 dB (SD = 3.67 dB) and 6.8 dB (SD = 3.4 dB) for the right and left eyes, respectively, demonstrating interocular symmetry. Coefficient of repeatability for overall mean sensitivity: <2 dB. Nine out of 10 subjects had a preserved inferotemporal subfield, whose mean sensitivity was highly correlated to the central field (r^2^ = 0.78, *P* = 0.002 and r^2^ = 0.72, *P* = 0.002 for the right and left eyes, respectively). Within the central field, sensitivities were greater in the temporal than the nasal half (*t*-test, *P* = 0.01 and *P* = 0.03 for the right and left eyes, respectively).

**Conclusions:**

Octopus static-automated perimeter demonstrates good repeatability. Interocular symmetry permits use of the noninterventional eye as an internal control. In this cohort, the inferotemporal and central visual fields are preserved into later disease stages likely mapping to populations of surviving cones.

**Translational Relevance:**

A consistently preserved inferotemporal island of vision highly correlated to that of the central visual field may have significance as a possible future therapeutic site.

## Introduction

*RPGR*-related retinitis pigmentosa (RP) is the most common cause of X-linked retinitis pigmentosa. The usual phenotype is of a rod-cone dystrophy, presenting with early onset nyctalopia and peripheral visual field loss that encroaches centrally toward the macula in early adult life. Often, there is relative preservation of a temporal island of visual field, which is common to many genetic etiologies of RP and may be important to retaining navigational function.^1^^–^^3^ The Goldmann kinetic perimetry is well-established in assessing the peripheral visual field. In subjects with *RPE-65* related retinal dystrophy treated with voretigene neparvovec, increases in the Goldmann visual field area were associated with improved performance in the multi-luminance mobility test (MLMT).^4^ However, the Goldmann perimeter is no longer in production.^5^ Automated or semi-automated kinetic perimetry, such as that obtained with the Octopus 900 perimeter (Haag-Streit AG, Koniz, Switzerland), demonstrates good qualitative agreement to the original Goldmann machine in patients with inherited retinal dystrophies.^6^ One advantage of such newer systems is that the visual field area is a default output, whereas Goldmann plots require manual treatment and digitization of the paper plots to provide similar data.

In contrast to kinetic perimetry, static automated perimetry measures retinal sensitivity, given in decibels, at a specific retinal location. Longitudinal assessment using the same testing grid pattern can measure localized changes in retinal sensitivity over time, either due to disease progression or in response to novel therapies. This is especially relevant when considering that gene-based treatments may be administered via a localized subretinal bleb. The precision of the perimeter can be increased by fundus tracking, such as in microperimetry, which is a key primary outcome in ongoing gene-replacement clinical trials phase I/II for *RPGR* related retinitis pigmentosa.^7^ Visual field areas can be assessed qualitatively via the default heatmaps, or quantitatively, such as through use of volumetric measures.^8^^,^^9^

Historically, however, static automated perimetry has not easily been applied to characterizing very peripheral function due to long testing times. The development of new testing algorithms, such as the German Adaptive Thresholding Estimation (GATE) strategy, uses several novel concepts, including termination of staircase testing at areas of deep scotoma which dramatically shorten test times and makes full-field perimetry a more feasible clinical test. This testing strategy has been validated against the current Swedish Interactive Thresholding Algorithm (SITA) gold standard.^10^ An important prospective study in patients with *RPGR*-related retinitis pigmentosa has already used wide-field testing grids on the Octopus 900 device to establish the degree of interocular symmetry and expected progression rates for overall mean sensitivity and volumetric measures.^8^ However, test-retest variability has not yet been reported for the mean sensitivity or volumetric measures, which would be required to define a statistically significant change in a cohort – for example, in the context of a clinical trial. Consequently, in this study, we aim to establish repeatability values for wide-field static automated perimeter using the Octopus 900 device, both for global measures and for the inferotemporal visual field. We also report on the phenotype in wide-field perimetry in *RPGR*-related RP – specifically the relation between central and peripheral photopic retinal sensitivity. This work aims to increase our understanding of not only the pattern but also the sensitivities in these regions via point sensitivities, mean thresholds, and volumetric measures, and repeatability within this patient cohort. Further studies such as these are paramount before any future considerations of potential therapy in these peripheral regions.

## Methods

### Clinical Assessment

A retrospective cross-sectional study of 10 male patients with confirmed pathogenic mutations in the *RPGR* gene were assessed as part of screening, but prior to recruitment to a phase I/II clinical trial of retinal gene therapy (ClinicalTrials.gov identifier NCT03116113). All participants provided informed consent and the work was conducted in accordance with the tenets of the Declaration of Helsinki. All patients undertook static automated perimetry performed with the Octopus 900 (Haag-Streit AG, Koniz, Switzerland), using a custom 185-point testing grid. The testing grid comprised a radial pattern extending 55.5 degrees nasally and superiorly, 67 degrees inferiorly, and 80 degrees temporally. Full threshold testing was performed using the GATE strategy^11^ with Goldmann size V stimuli against a photopic background luminance of 31.4 apostilbs (10 candela/m^2^). The GATE strategy has the capability to use thresholds obtained in previous examinations in order to inform subsequent tests and therefore shorten testing times. This latter feature, however, was not used in our study, with follow-up tests recorded as new examinations, in order to ensure that increasingly shorter examination times did not bias our determination of test-retest repeatability. The test was divided into two stages. In stage 1, stimuli were presented in the central 30 degrees and with corrective lenses in place. Once completed, the testing sequence automatically pauses, and the corrective lenses were automatically removed. In stage 2, more peripheral stimuli were presented. To minimize fatigue, patients were permitted a short break of approximately 5 minutes during this pause, and were also given verbal encouragement by the examiner throughout testing. Tests were performed monocularly with the right eye tested first. Subjects underwent triplicate testing across a 2-day period. Test reliability was determined by the reliability factor (RF), which is a standard output of the Octopus 900 perimeter and given by the sum of false-positive and false-negative scores divided by the total number of positive and negative catch trials presented. For the purposes of this study, results from tests with RF scores of greater than 25% were excluded to avoid significant positive or negative bias to the mean sensitivity.^8^ To our knowledge, the effects of false-positive and false-negative catch trials in inherited retinal degenerations have not been specifically studied. The upper limit of 25% has been used in prior studies of perimetry in inherited retinal diseases to good effect.^8^^,^^12^^,^^13^ Prior to perimetric testing, patients also underwent subjective refraction and measurement of best corrected visual acuity using Early Treatment Diabetic Retinopathy Study (ETDRS) charts placed at 4 meters.

### Visual Field Analysis

**Figure 1.**
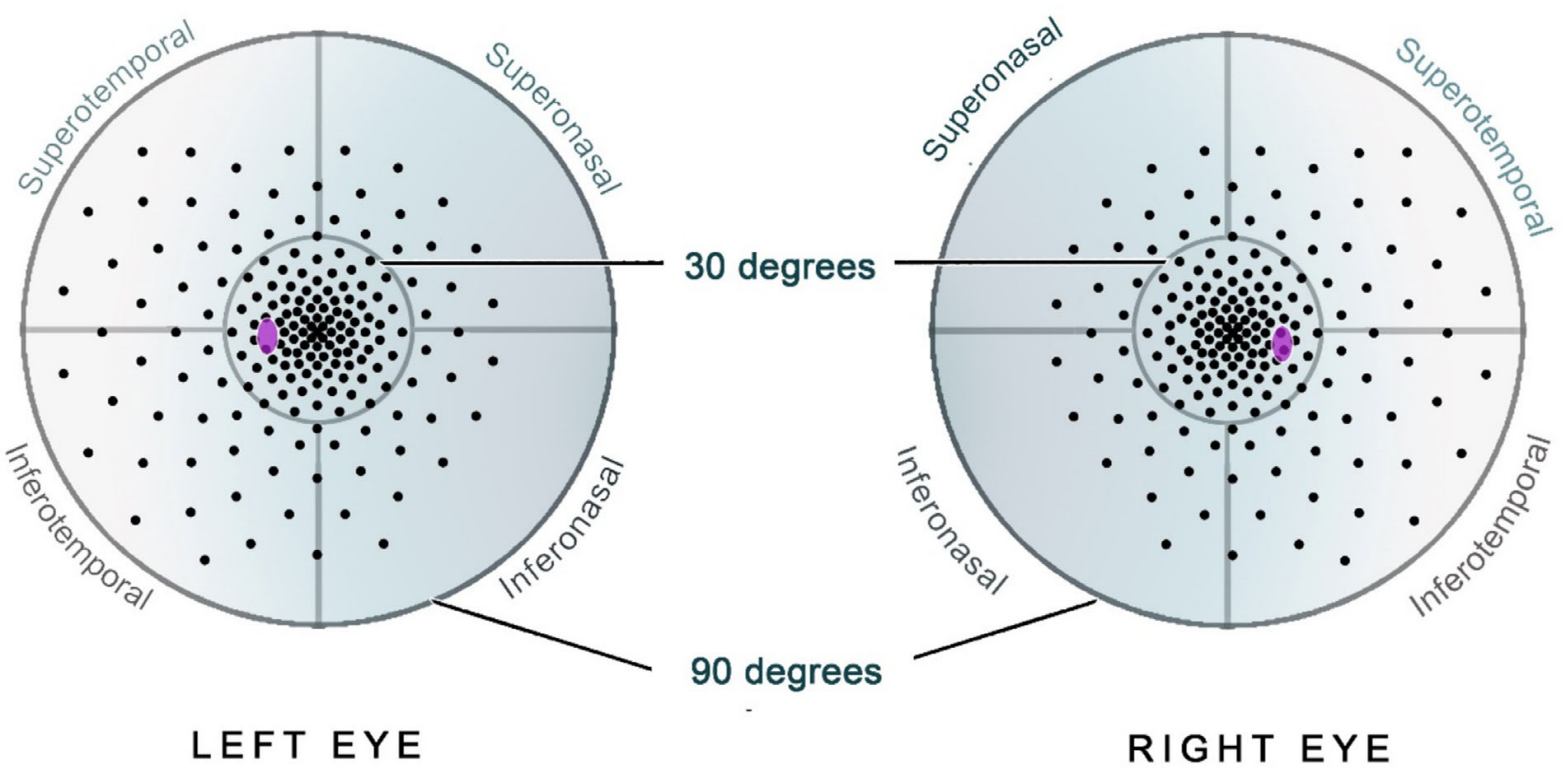
Schematic of subfield definitions. *Black points* represent the 185-point testing grid. The central 30-degrees is within the *innermost circle*. The blind spot is highlighted in *purple*. Number of points in each subfield are as follows: central 30 degrees (*n* = 108); inferotemporal (*n* = 22); inferonasal (*n* = 17); superotemporal (*n* = 22); and superonasal (*n* = 16).

The overall mean sensitivity is a standard Octopus output, calculated from the mean sensitivity of individual thresholded points (referred to as pointwise data) across the testing grid. There exists some discussion on the validity of averaging a logarithmic scale, such as decibels, and whether functional measures are best transformed to linear scales (candela per meter squared for perimetric stimuli).^14^ For the purposes of this study, we have performed averaging on the decibel scale to be consistent with the Octopus standard output. Export of the pointwise data was used to subdivide the visual field into five areas of interest – herein referred to as subfields (Fig. 1^2^). These subfields were as follows: the central radius of the 30 degrees field, representing the central retinal sensitivity, and peripheral superonasal, superotemporal, inferonasal, and inferotemporal subfields (see Fig. 1). Mean sensitivity per subfield was calculated by the geometric mean of the decibel point sensitivity values within each subfield. Raw decibel pointwise data were used to create composite visual field heatmaps across the cohort for the full-field grid and the central 30-degree subfield using linear interpolation in R programming language within the Akima package.^15^

**Figure 2.**
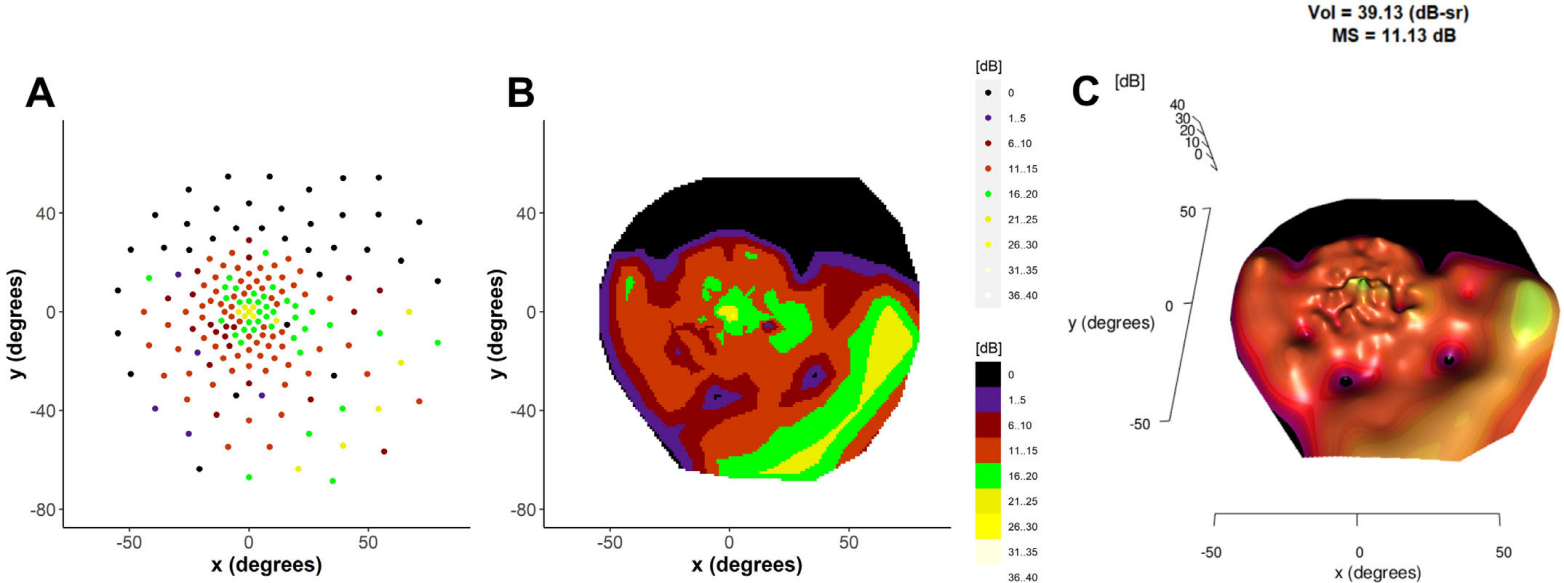
Example of the generation of a volumetric measure of the hill of vision. (**A**) Point map of raw static perimetry data. (**B**) Interpolated data allowing the generation of heat maps. (**C**) Representation of the decibel sensitivities in the z-plane resulting in a 3-dimensional hill of vision with corresponding volume metric.

### Interocular Symmetry and Repeatability Analyses

Interocular symmetry was calculated for overall mean sensitivity and all subfields by bivariate correlation, with Pearson's correlation coefficients in the case of normally distributed data and Spearman's ρ analyses for non-parametrically distributed data. Test-retest repeatability was defined in the mean sensitivities, pointwise sensitivities, and volumes by calculation of coefficients of repeatability and limits of agreement, as per the method given by Bland and Altman.^16^ Pointwise variability was calculated accounting for floor effects by excluding any test pairs with one or both results of 0 dB (i.e. insensitive).^17^^,^^18^ Ceiling effects were not considered relevant, as the Octopus has a wide dynamic range of stimulus intensities, the limit of which was not approached by the cohort.

### Volumetric Analyses

Aggregated pointwise sensitivities obtained from static perimetry can be interpreted by a simple mean of the point values or by consideration of the volume under a hill of vision. The hill of vision is a long standing concept which has recently been applied to modern static perimetry resulting with several advantages; an accurate assessment of total or regional sensitivities taking variable grid spacing into account, avoidance of artificially deflating regional or overall mean sensitivities in the presence of a significant number of zero pointwise values; and a graphical representation of the hill of vision allowing for a more intuitive visualization of retinal function (Fig. 2). In this study, we use a custom program adapted from previous work on the re-interpretation of microperimetry results.^9^ The article contains a detailed description of the method and open-source code for microperimetry application.

### Statistical Analyses

Statistical analyses were conducted with SPSS (version 26.0; IBM Software, Armonk, NY) and in R (version 3.6.3)^19^ with figures produced using the package ggplot2.^20^ Normality of all data was verified utilizing Shapiro-Wilk analyses prior to subsequent correlations. Bonferroni post hoc correction was applied in the case of multiple comparisons.

## Results

### Patient Characteristics

**Figure 3.**
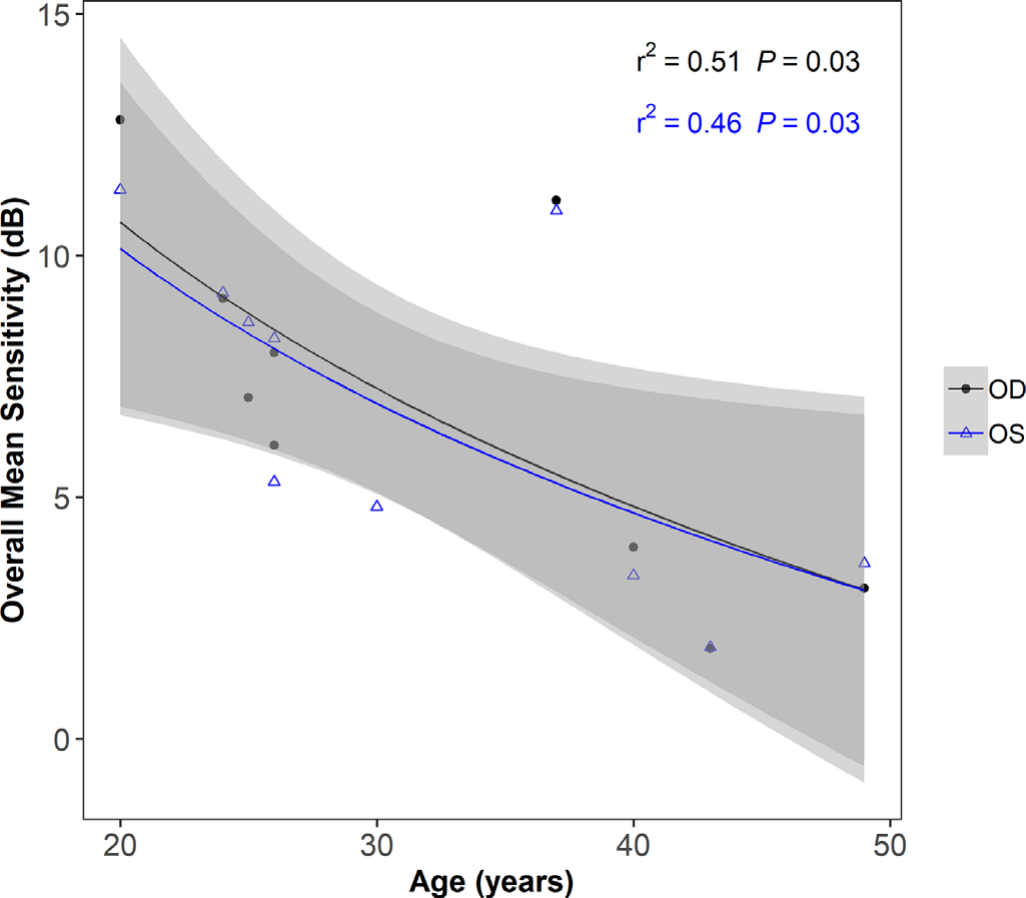
Age versus overall mean sensitivity (*n* = 9 for the right eye, and *n* = 10 for the left eye). *Shaded areas* show 95% confidence limits of regression fit. *Black and blue points* and regression lines are for the right (OD) and left (OS) eyes, respectively.

The mean patient age was 32 years (range = 20 to 49 years, SD = 9.6 years), with a mean best corrected visual acuity of 0.30 logMAR (range = 0.00 to 0.80, SD = 0.24 logMAR) and 0.25 logMAR (range = −0.04 to 0.48, SD = 0.14 logMAR) in the right and left eyes, respectively. Median testing time was 18.23 minutes. Patients were typically myopic with a mean best spherical equivalent of −2.23 diopters (D); SD = 3.80) and −2.13 D (SD = 3.23) in the right and left eyes, respectively (Table 1). Overall, the mean sensitivity was correlated to the patient's age (Fig. 3) and was best described by a logarithmic function. Logarithmic regression, Pearson's *r* squared correlation coefficient, *r*^2^ = 0.52 and 0.47; *P* values *P* = 0.028 and *P* = 0.028 for the right and left eyes, respectively.

**Table 1.** Visual Acuity, Subjective Refraction, and Mean Sensitivity of Individual Subjects

	*OD*				*OS*			
Subject Number	Best Vision Sphere (D)	BCVA (logMAR)	OMS (dB)	V_Tot_ (dB-sr)	Best Vision Sphere (D)	BCVA (logMAR)	OMS (dB)	V_Tot_ (dB-sr)
1	0.25	0.80	1.87	2.56	−0.25	0.48	1.92	2.1
2	−2.50	0.26	**^a^**	—**^a^**	−2.0	0.22	4.82	18.14
3	−5.50	0.14	7.06**^a^**	20.02**^a^**	−5.75	0.16	8.23	26.12
4	1.25	0.08	12.81	41.06	1.50	0.20	11.33	37.6
5	−1.0	0.52	3.97	3.04	−0.50	0.40	3.42	2.5
6	0.0	0.20	3.11	5.44	0.00	0.22	3.67	6.01
7	−4.25	0.00	9.11	33.73	−4.25	−0.04	9.30	33.87
8	−2.25	0.22	7.99	22.86	−2.00	0.22	8.36	25.87
9	0.75	0.40	6.07	12.27	0.75	0.32	5.38	10.06
10	−9.00	0.42	11.14	39.13	−8.75	0.34	11.05	38.44

a Exclusion of at least one unreliable test (reliability factor >25%, *n* = 5). Additionally, subject 2 had three unreliable tests in the right eye and therefore no mean sensitivity is calculated.

Here, overall mean sensitivity (OMS) of test three from triplicate testing is reported unless this test result was deemed unreliable (reliability factor >25%).

D = diopters, BCVA = best corrected visual acuity.

### Repeatability and Interocular Symmetry

Five tests (4 of which were in one subject) had an RF >25% and were excluded. Analyses were performed on the remaining 55 tests. Individual visual acuity and mean sensitivities are given in Table 1. The overall mean sensitivity was highly correlated between the right and left eyes (*r* = 0.97, *P* < 0.001), as well as across the five individual subfields (Table 2). The limits of agreement tended to be narrower between test 2 and test 3 for overall mean sensitivity and pointwise sensitivity compared to that between test 1 and test 2, indicating that a learning effect had taken place (Table 3).^21^ However, test-retest variability did not appear to improve throughout repeat resting for the inferotemporal volumetric sensitivity.

**Table 2.** Interocular Symmetry for Subfield Sensitivities, Across the Cohort

	Categorical Mean Values (dB) (SD)				Test of Normality Using Shapiro-Wilk (*P* Value)	
	OD	OS	Mean Interocular Difference (dB) (SD)	Interocular Symmetry – Correlation Coefficients (Pearson's *r* or Spearman ρ) (*P* Value)	OD	OS
**Overall MS (dB)**	7.0 (3.7)	6.8 (3.4)	0.6 (0.6)	r = 0.97 (*P* < 0.001)	*P* = 0.92	*P* = 0.44
**Central 30 degrees MS (dB)**	9.5 (4.1)	8.8 (3.7)	0.7 (0.7)	r = 0.97 (*P* < 0.001)	*P* = 0.96	*P* = 0.39
**Inferior temporal MS (dB)**	6.1 (4.9)	6.0 (4.6)	0.9 (1.0)	r = 0.96 (*P* <0.001)	*P* = 0.65	*P* = 0.23
**Superior temporal MS (dB)**	3.5 (3.4)	3.7 (3.0)	1.2 (1.0)	r = 0.88 (*P* = 0.002)	*P* = 0.18	*P* = 0.48
**Inferior nasal MS (dB)**	2.8 (3.6)	2.6 (3.6)	1.0 (1.4)	ρ = 0.86 (*P* = 0.003)^a^	*P* = 0.008^a^	*P* = 0.005^a^
**Superior nasal MS (dB)**	1.3 (1.9)	1.9 (2.5)	0.9 (1.1)	ρ = 0.8 (*P* = 0.01)^a^	*P* = 0.002^a^	*P* = 0.007^a^

The overall mean and individual sub-field sensitivity values given are for test 3 where this was deemed reliable (reliability factor ≤25%). Where no reliable result in test 3 was available, the results from test 2 or test 1 was used. If reliability factor >25% for all tests, the patient results were excluded from all subsequent analysis.

a Non-normally distributed data, requiring Spearman correlation.

MS = mean sensitivity.

**Table 3.** Coefficients of Repeatability and Limits of Agreement for Overall Mean Sensitivity, Pointwise Sensitivity; Inferotemporal Mean Sensitivity; Total Sensitivity Volume (Vtot); Central 30 Degree Volume Sensitivity (V30); and Inferotemporal Sensitivity Volume (V. Inferotemp) Between Test 2 and Test 1; and Test 3 and Test 2

	Test 2 – Test 1				Test 3 – Test 2			
	OD		OS		OD		OS	
	CoR	Limits of Agreement	CoR	Limits of Agreement	CoR	Limits of Agreement	CoR	Limits of Agreement
*Overall mean sensitivity (dB)*	2.41	−2.77 to 2.06	1.80	−2.36 to 1.24	1.72	−1.23 to 2.21	0.96	−0.70 to 1.24
*Inf temp mean sensitivity (dB)*	4.61	−4.01 to 5.2	3.44	−4.35 to 2.52	2.18	−1.82 to 2.54	3.55	−3.2 to 3.9
*Vtot (dB-sr)*	9.96	−12.1 to 7.82	7.24	−9.48 to 5	6.42	−4.38 to 8.45	6.89	−6.07 to 7.71
*V30 (dB-sr)*	1.78	−2.04 to 1.52	1.57	−2 to 1.13	1.65	−1.15 to 2.15	1.06	−0.72 to 1.39
*V_inftemp (dB-sr)*	3.96	−4.17 to 3.75	3.45	−4.32 to 2.57	2.18	−1.75 to 2.6	4.56	−4.18 to 4.94
*Pointwise sensitivity (dB)*	9.64	−9.91 to 9.36	9.39	−9.98 to 8.81	9.23	−8.76 to 9.69	8.62	−8.38 to 8.87

CoR = coefficient of repeatability, dB = decibels, dB-sr = decibel steradians.

### Volumetric Versus Mean Sensitivity Repeatability Analysis

**Figure 4.**
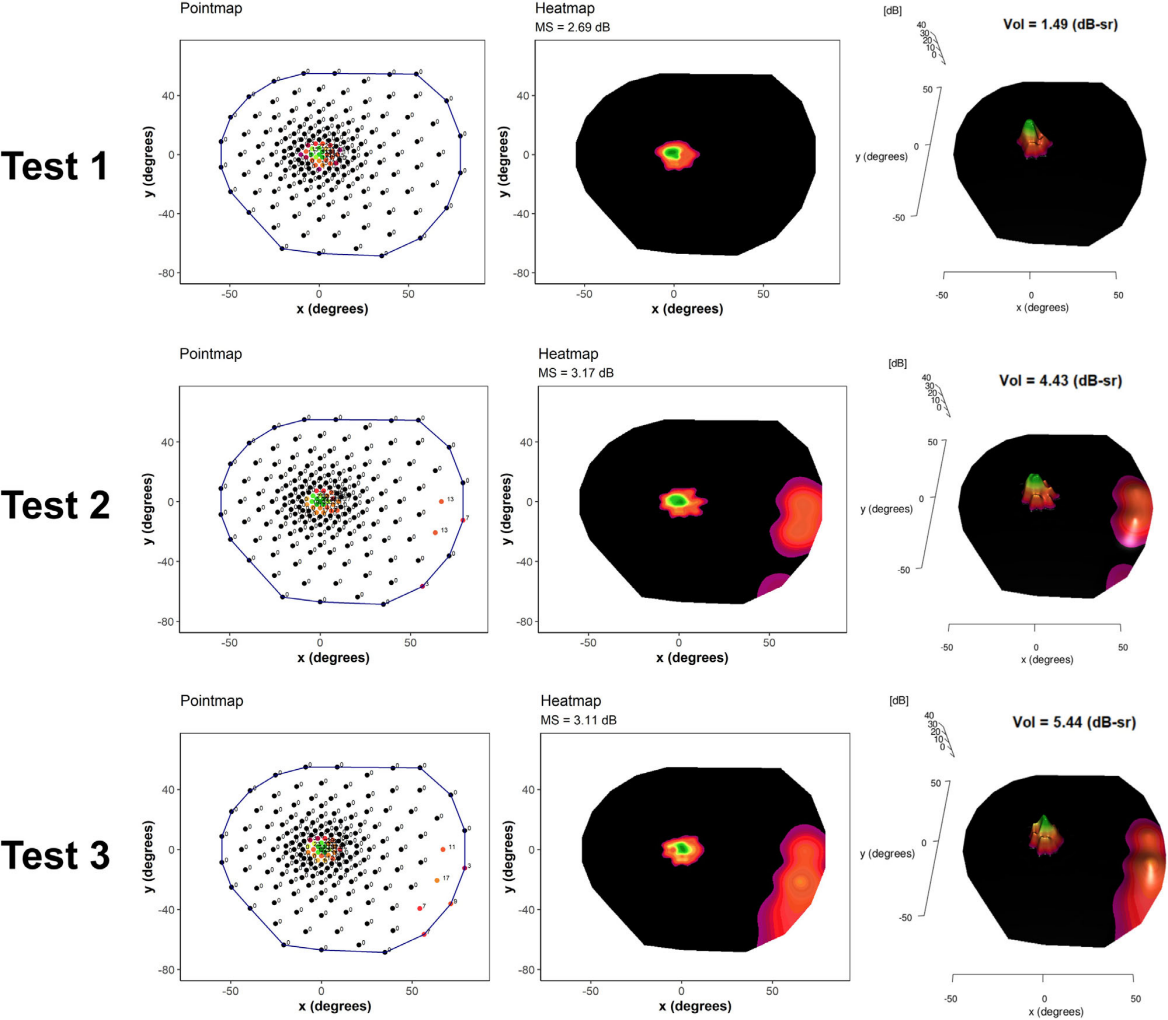
Example patient results across triplicate testing shown with point maps on the left, heat maps in the center and 3D volumetric plots on the right. Tests 1, 2, and 3 are labeled to show the order in which triplicate testing was performed over two consecutive days. dB-sr = decibel-steradians.

One significant drawback to the use of mean sensitivity as an overall measure of function is its validity of use in grid patterns where non-uniform spacing are used.^9^ In these instances, mean sensitivity measures are heavily weighted in favor of the densely sampled central region and underweighted for more sparsely tested peripheral regions, as is routinely the case in full field static perimetry. Threshold values in the peripheral regions contribute relatively little to the overall average of point sensitivities across the whole field. Volumetric measures do not suffer from this weighting issue as regions between points are interpolated and volumes are a product of spatial extent as well as decibel values. The presence of extensive scotoma in RP is also relevant as the averaging of zero-decibel values greatly depresses the mean. As volume is not an average measure, but rather an additive one, this problem is avoided. The implications of this are demonstrated in the triplicate testing of one of our patient case examples shown in Figure 4. Here, in test 1, there was no demonstration of peripheral island, possibly due to learning effects or patient fatigue during the test. In test 2 and test 3, the peripheral island is evident, and despite the relatively low thresholds in these regions contributing little to the overall mean sensitivity, the volumetric measures take into account the large spatial area of these regions and so contribute significantly to the overall volume. We also note that despite the mean sensitivity reducing between test 2 and test 3, the volume actually increases. This exemplifies the difference between the two measures in the mean sensitivity being greatly affected by the extent of the scotoma, whereas the volume is not affected in the same way.

### Characterizing the Peripheral Visual Field in RPGR-Related RP

**Figure 5.**
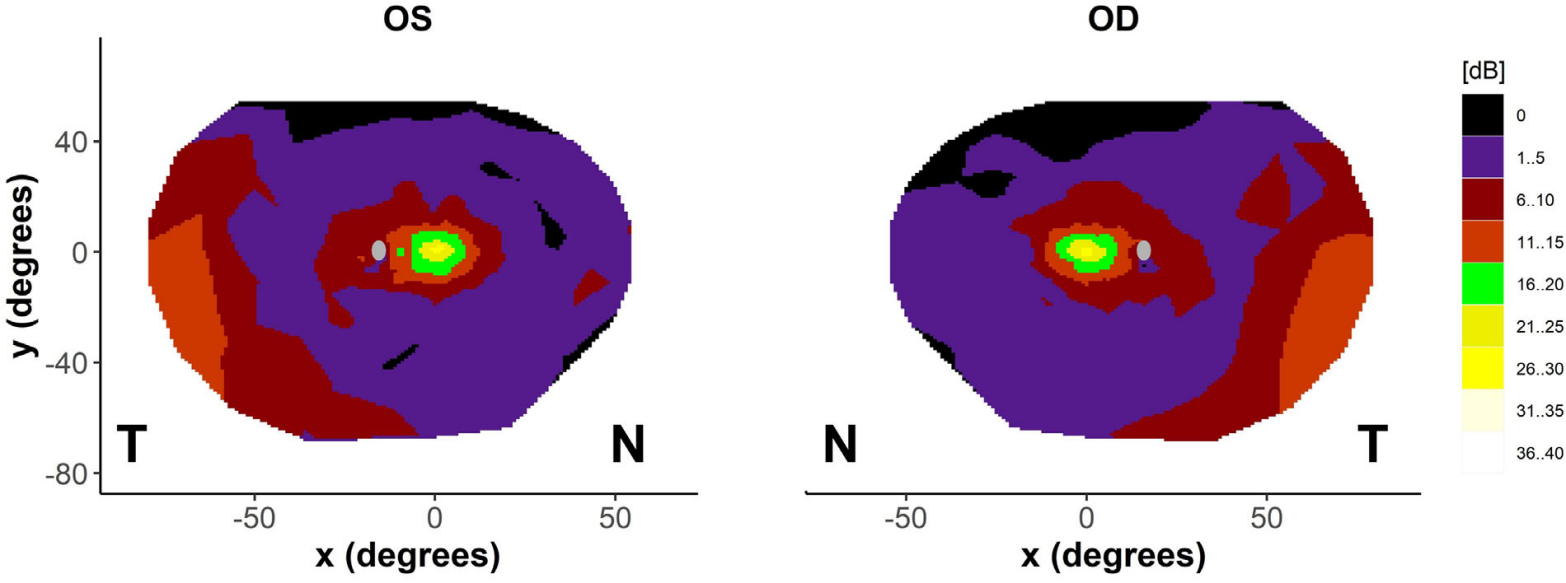
Composite visual fields (full field) for all patients (*n* = 9 for the right eye, and *n* = 10 for the left eye) demonstrating the majority of patients have well preserved central and inferotemporal visual fields. Scotoma across superior fields likely due to eyelid effects. N = nasal, T = temporal.

**Figure 6.**
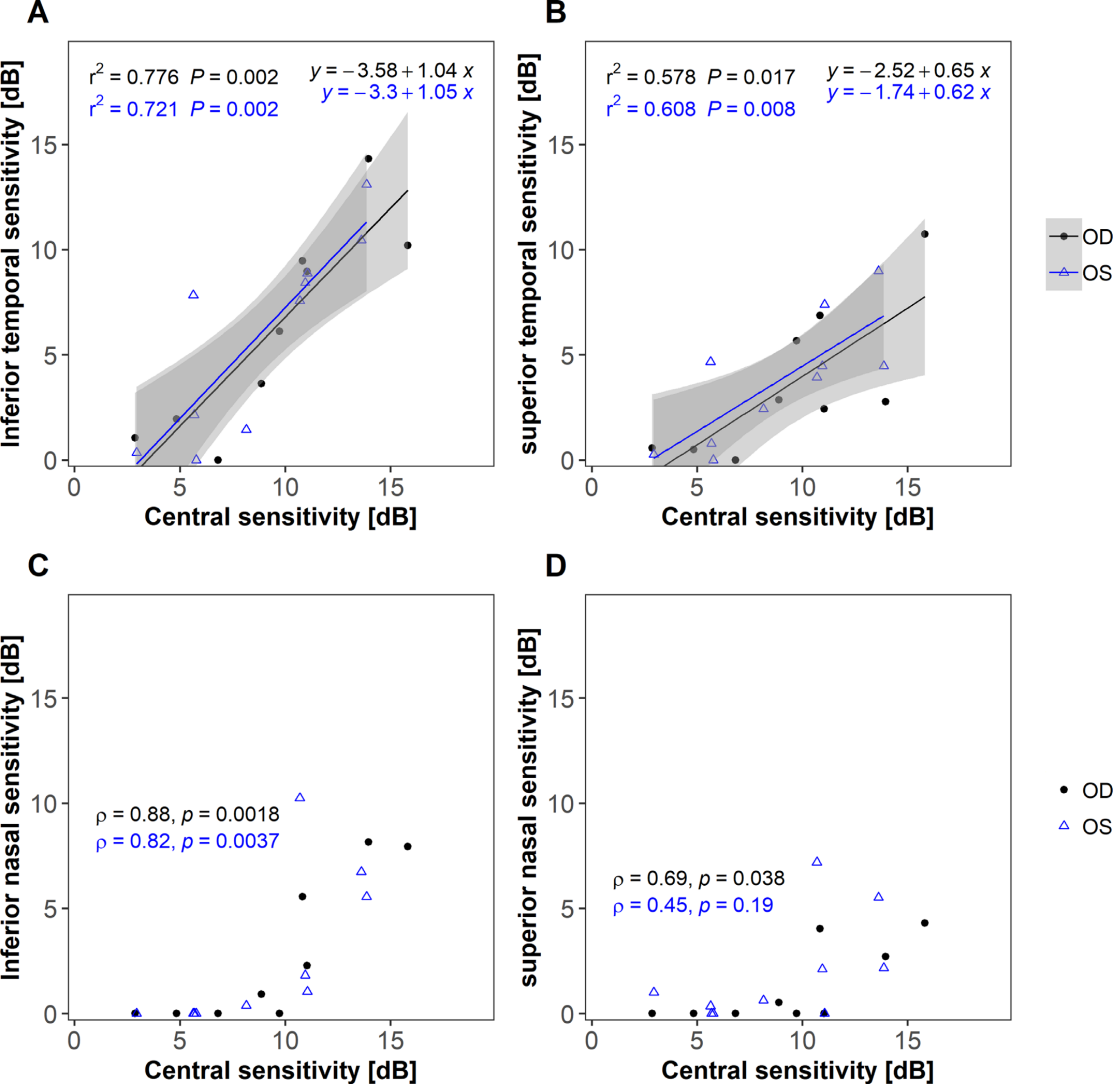
Correlations between mean sensitivity of the central 30-degrees. (**A**) Inferior temporal sensitivity, (**B**) superior temporal sensitivity, (**C**) inferior nasal sensitivity, and (**D**) superior nasal sensitivity (*n* = 10). *Shaded areas* show 95% confidence limits of regression fit. *Black and blue points* are for the right and left eyes, respectively. Bonferroni significance level is *P* = 0.0125. Nasal quadrants are dominated by floor effects whereby majority of subjects had little to no sensitivity.

**Figure 7.**
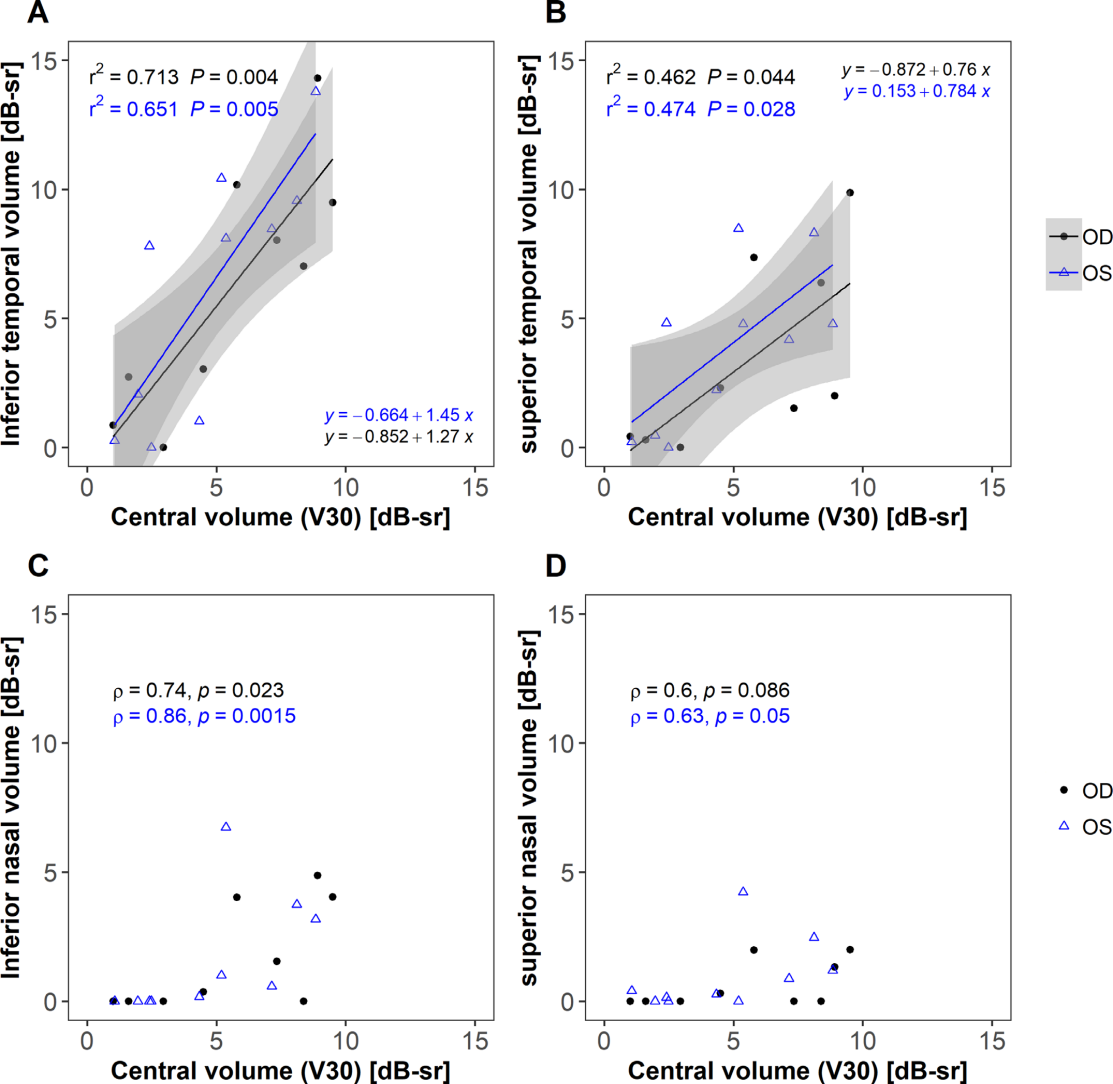
Correlations between volume of the central 30-degrees (V30). (**A**) Inferior temporal volume, (**B**) superior temporal volume, (**C**) inferior nasal volume, and (**D**) superior nasal volume (*n* = 10). Units of volume in decibel steradians (dB-sr). *Shaded areas* show 95% confidence limits of regression fit. *Black and blue points* are for the right and left eyes, respectively. Bonferroni significance level is *P* = 0.0125.

In this cohort, 9 out of 10 patients demonstrated measurable sensitivity in the inferotemporal quadrant, which was absent in one subject in their 40s. Decibel pointwise sensitivities for each location were averaged across all patients and a composite sensitivity heatmap using linear interpolation for the entire cohort of patients across the full field is shown in Figure 5. The sensitivity of the central 30 degrees and that of the inferotemporal visual field were highly correlated (*P* = 0.002; Fig. 6A), with the gradient of the fitted regression line close to 1. The sensitivity of the superotemporal quadrant was also correlated to that of the central 30 degrees (Fig. 6B), although with marked difference in gradient to the inferotemporal quadrant. A similar pattern is observed with volumetric measures but with a steeper gradient in Figure 7A likely representing a significant decline in the remaining area as well as sensitivity thresholds compared to the central region. A significant proportion of subjects had low or no sensitivity in the inferonasal and superonasal quadrants with little to no measured sensitivity in the inferonasal or superonasal fields in subjects with central sensitivities of less than 10 dB (Figs. 6C, 6D). A range of fitting functions were attempted along with the exclusion of those patients in which the zero decibel floor effect was reached, however, the significant number of zero decibel values makes the fitting of any regression line questionable, so it has been excluded in favor of presenting the raw data points.

### Nasal versus Temporal Asymmetry Within the Central Visual Field in *RPGR*-Related RP

**Figure 8.**
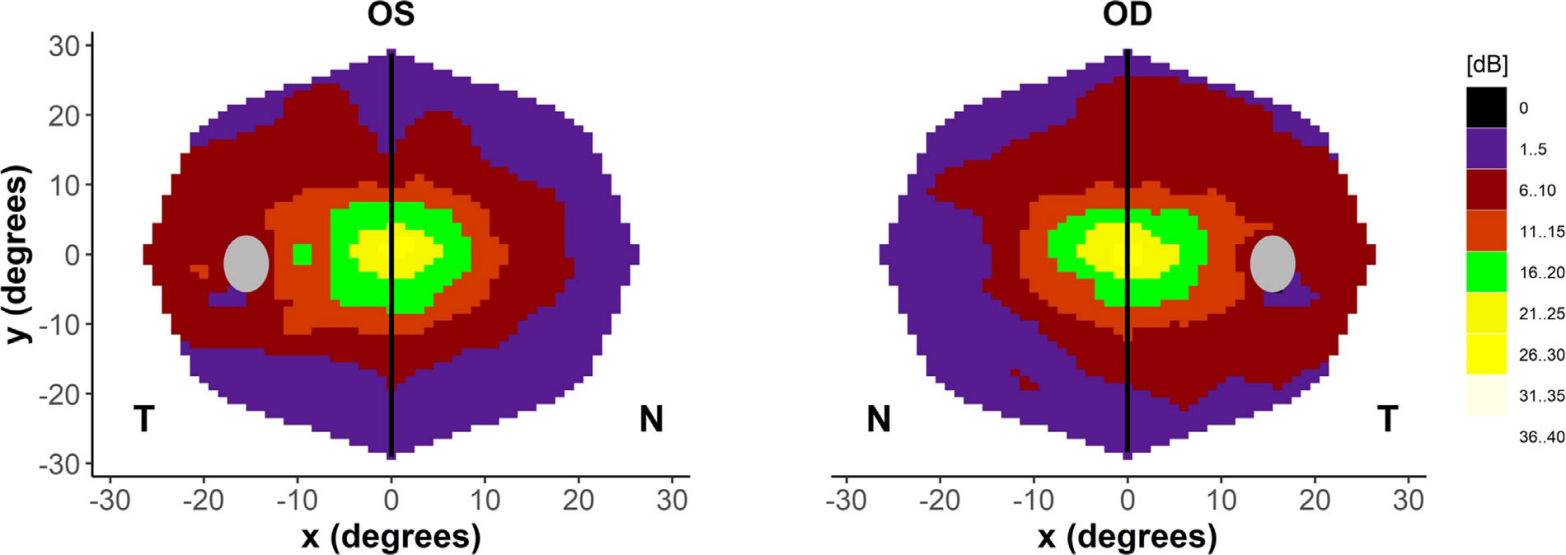
Nasal-temporal asymmetry within the central visual field. Composite heatmap for our cohort of patients with *RPGR*-related RP (central 30-degree field; *n* = 9 for the right eye, and *n* = 10 for the left eye) demonstrating nasal-temporal asymmetry in retinal sensitivity. There were 47 points that were included in each hemifield due to exclusion of points lying directly on the vertical meridian, and the corresponding points in the nasal hemifield to account for the blind spot. Grey oval = blind spot, N = nasal, T = temporal.

Within the central 30 degrees of visual field, we observed relative preservation of the temporal hemifield around the physiological blind spot (Fig. 8). The geometric mean of each point sensitivity across all subjects was calculated within the central nasal and central temporal hemifields. Subsequently, the average hemifield sensitivity was calculated from the geometric mean of the averaged pointwise sensitivity at each location (see Fig. 8). The mean difference between the central nasal and temporal hemifields (temporal minus nasal) was 2 dB (SD = 1.85, SEM = 0.62) for the right eye and 1.9 dB (SD = 2.2, SEM = 0.73) in the left eye, which was statistically significant (paired *t*-test, *P* = 0.01 and *P* = 0.03 for the right and left eyes, respectively).

## Discussion

In this cross-sectional observational study, we establish repeatability values for wide-field static automated perimetry using the Octopus 900 device in a group of patients with *RPGR*-related RP. As perimetry was obtained using a photopic background illumination of 10 cd/m^2^, the distinctive visual field patterns observed are in agreement with previous observations and are hypothesized to be due to surviving cone populations across the retina.^22^^,^^23^ We demonstrated that the photopic sensitivities in the central and inferotemporal fields are highly correlated, which expands on the knowledge that a peripheral island is maintained. The gradient of the fitted regression line (see Fig. 6A) is close to 1, demonstrating a close relationship in the mean sensitivity between these 2 regions in the cohort, with a steeper gradient in volume decline (see Fig. 7A) indicating that area loss as well as threshold reduction is likely to play a significant role in peripheral decline compared to that of the central region. Although most patients retained inferotemporal sensitivity, fewer patients retained superotemporal sensitivity, and fewer still the infero- and supero-nasal quadrants. This observation may be related to topographical variation of cone densities across the healthy retina. It is known from histological studies in normal eyes that the superonasal retina is highly enriched in cones in comparison to other areas^22^^,^^24^^,^^25^ giving rise to the inferotemporal visual field. Charng et al.^23^ used wide-field dark-adapted and light-adapted chromatic perimetry to define rod and cone sensitivity losses across the retina in a cohort of patients with *RPGR*-related RP. The majority of the cohort retained sensitivity in the inferotemporal quadrants that was cone mediated. In a subset of patients, longitudinal data suggest nasal-temporal differences in the rate of cone degeneration, with loci responsible for supero- and inferonasal visual fields losing sensitivity at a greater rate than those in the temporal periphery. This has also been replicated in a subsequent study in 15 subjects with *RPGR*, demonstrating higher rates of rod and cone-mediated sensitivity losses in nasal versus temporal subfields.^26^ These fields were selected on a per-patient basis but concentrated on sites within the central visual field. Consistent with these studies, we demonstrate nasal-temporal asymmetry in the central visual field in our cohort, with greater sensitivity surrounding the physiological blind spot. In vivo preservation of autofluorescence signal (using wide-field imaging) can be observed at the nasal peripapillary border in patients with RP.^27^ In healthy controls, adaptive optics imaging up to 24 degrees of eccentricity on the horizontal meridian demonstrates an increase in cone density around the disc, being approximately 34% greater nasally than temporally.^28^ If photoreceptor death is a fixed probability over time,^29^ higher regional cellular densities are expected to result in a slower rate of functional sensitivity losses. However, as localized cone and rod-mediated sensitivity losses, as well as overall disease severity and phenotype, may vary even in individuals with the same mutation,^30^ this implies other genetic determinants of photoreceptor degeneration likely interact with underlying photoreceptor topography.^23^

Defining test-retest variability is of critical importance for both observational and interventional studies. Our pointwise coefficient of repeatability is in broad agreement with that reported by Bittner et al. – 8.74 dB – using a Humphrey device and the same size stimulus (with the exclusion of “low sensitivity points”).^31^ Previously, Tee et al. calculated an annual decline of 0.69 dB per year in the mean sensitivity in a prospective study of *RPGR-*related patients with RP under similar testing conditions.^8^ Based on our calculated coefficients of repeatability, the minimum interval between examinations to detect a statistically significant change in the overall mean sensitivity in our cohort would be approximately 2.5 years. This may facilitate planning of a clinical follow-up. In addition to the overall mean sensitivity, we define repeatability for individual test points. Like microperimetry, the pointwise variability is greater than that of the overall mean sensitivity^32^; however, outcomes based on points or clusters of points may be required to detect the effects of locally applied treatments (for example, via a subretinal bleb) which would not be expected to change the mean sensitivity across the whole visual field, especially when this is biased due to floor effects from scotoma. Whereas a learning effect was apparent for some indices, there was no observed improvement in test-retest variability in the inferotemporal volume. Figure 4 illustrates the potential role of learning effects or patient fatigue, in that during the first test, there was no evidence of inferotemporal sensitivity but sensitivity in this region was evident on subsequent testing. The median test time is 18 minutes, with the peripheral retina tested after the central retina. In order to reduce test-retest variability, and decrease the likelihood of a type II error in an interventional trial, it may be beneficial to trial alternate test grids that omit testing of the central region and sample the peripheral retina of interest more densely or consider a significantly longer break between central and peripheral testing regimes.

The marked interocular symmetry in perimetric indices across the cohort is in agreement with previously published data for Octopus perimetry in *RPGR*-related patients with *RPGR*-related RP and other visual function tests, such as visual acuity, microperimetry,^32^ and the Goldmann visual field areas.^33^ Consequently, in the context of an interventional trial, the fellow eye may serve as the control. Further investigation is required to determine optimum surgical technique to access the peripheral subretinal space.^34^^–^^36^

A limitation to our analysis is that its cross-sectional nature precludes independent verification of reported progression rates in the loss of retinal sensitivity across the visual field. A further limitation is the relatively small sample size examined. In order to fully determine patterns of field preservation in *RPGR*-related RP beyond this cohort of patients, a larger prospective study would be required to achieve sufficient statistical power.

In conclusion, this analysis characterizes patterns of visual field loss with full-field static automated perimetry in a cohort of patients with *RPGR*-related RP. We demonstrate that the test is reproducible and this cohort demonstrates a high degree of interocular symmetry required for interventional studies in which the fellow eye serves as the control. We observe preservation of the inferotemporal peripheral field along with greater central temporal retinal sensitivity, that remain spared until the later disease stages. These data may inform future research into observational or interventional studies that focus on the peripheral visual field which is critical to navigational vision.

### Ethics Oversight

The study was conducted with approval from South Central – Oxford A Research Ethics Committee (REC reference 16/SC/0551). The study was conducted in full conformity with all applicable laws and regulations, including the International Conference on Harmonisation Guidelines for Good Clinical Practice (CPMP/ICH/135/95) and relevant articles of the Declaration of Helsinki (seventh revision, 2013). Written informed consent was obtained from each study participant.
